# An Evaluation of Patient-Physician Communication Style During Telemedicine Consultations

**DOI:** 10.2196/jmir.1193

**Published:** 2009-09-30

**Authors:** Zia Agha, Debra L Roter, Ralph M Schapira

**Affiliations:** ^5^Milwaukee Veterans Affairs Medical CenterMilwaukeeWIUSA; ^4^Division of Pulmonary and Critical Care MedicineMedical College of WisconsinMilwaukeeWIUSA; ^3^Department of Health Policy and ManagementJohns Hopkins School of Public HealthBaltimoreMDUSA; ^2^Department of MedicineUniversity of CaliforniaSan DiegoCAUSA; ^1^Veterans Affairs San Diego Health Services Research and DevelopmentSan DiegoCAUSA

**Keywords:** Telemedicine, remote consultation, physician-patient relations

## Abstract

**Background:**

The quality of physician-patient communication is a critical factor influencing treatment outcomes and patient satisfaction with care. To date, there is little research to document the effect of telemedicine (TM) on physician-patient communication.

**Objective:**

The objectives of this study are to measure and describe verbal and nonverbal communication during clinical TM consultations and to compare TM with in-person (IP) consultations in terms of the quality of physician-patient communication.

**Methods:**

Veteran patients (n = 19) requiring pulmonary medicine consultations were enrolled into the study. The study group included 11 patients from the Iron Mountain Veterans Affairs Hospital (VAMC) remote site. Patients had individual TM consultations with a pulmonary physician at the Milwaukee VAMC hub site. A control group of 8 patients had IP consultations with a pulmonary physician at the Milwaukee VAMC. Video recordings of medical consultations were coded for patient-physician verbal and nonverbal communication patterns using the Roter Interaction Analysis System (RIAS).

**Results:**

There were no differences in the length of TM consultations (22.2 minutes) and IP consultations (21.9 minutes). Analysis of visit dialogue indicated that the ratio of physician to patient talk was 1.45 for TM and 1.13 for IP consultations, indicating physician verbal dominance. Physicians were more likely to use orientation statements during IP consultations *(P* = *.*047). There were greater requests for repetition from patients during TM consultations (*P* = .034), indicating perceptual difficulties.

**Conclusions:**

The study findings indicate differences between TM and IP consultations in terms of physician-patient communication style*.* Results suggest that, when comparing TM and IP consultations in terms of physician-patient communication, TM visits are more physician centered, with the physician controlling the dialogue and the patient taking a relatively passive role. Further research is needed to determine whether these differences are significant and whether they have relevance in terms of health outcomes and patient satisfaction with care.

## Introduction

Physician-patient communication problems are a common cause for patient dissatisfaction during in-person (IP) consultations. Dissatisfied patients are less likely to return for physicians’ appointments, more likely to switch physicians, and more likely to be noncompliant with recommendations [1]. Such nonadherence contributes to unnecessary diagnostic testing, results in potentially harmful regimen changes, and leads to wasted health resources [2-4].

Clinical telemedicine (TM) includes applications that use communication technologies to link specialists at a tertiary center to patients and primary providers at a remote site. It is unclear how the physical separation between patient and physician inherent in TM consultations affects physician-patient communication. A number of comprehensive reviews have identified the need to evaluate physician-patient interactions during TM consultations [5-7].

One objective of this pilot study is to describe the use of the “Roter Interaction Analysis System” (RIAS) as a comprehensive content coding methodology for assessing verbal and nonverbal communication during clinical TM consultations [1,8,9]. A second objective is to identify potential differences in physician-patient communication style during TM compared to IP consultations.

## Methods

### Study Setting and Design

We conducted an observational pilot study to compare the pattern of verbal and nonverbal physician-patient communication during TM and IP consultations. The study was conducted at the Milwaukee VAMC and was approved by the Institutional Review Board. The Milwaukee VAMC, a teaching affiliate of the Medical College of Wisconsin, is a tertiary care facility providing a full range of primary care and specialty services for veterans residing in the greater Milwaukee area. The Milwaukee VAMC provides TM services in medical subspecialties (eg, pulmonary medicine, rheumatology, and infectious disease) to the Iron Mountain VAMC in Michigan [10,11]. Our study population was drawn from a group of veterans referred for pulmonary medicine consultations. Subjects in the TM group (n = 11) were referred for TM consultation from the Iron Mountain VAMC. Those in the control group (n = 8) were referred for pulmonary IP consultation from the Milwaukee VAMC. Informed written consent was obtained from all subjects.

### Participating Physicians and Nurses

In the present study, 3 pulmonary specialists participated in conducting TM and IP consultations at the Milwaukee VA. These board certified specialists each had at least two years of experience in conducting TM consultations. To control for physician variability, the same group of physicians (n = 3) conducted both the IP and TM consultations. One of two nurses was always present during the TM consultations at the Iron Mountain remote site. These nurses are trained to assist with TM examinations and are familiar with the equipment and technology used during TM.

### Telemedicine Consultation Process

TM consultations were performed between the pulmonary specialists located at the Milwaukee VAMC telemedicine site and the patient and trained nurse located at the Iron Mountain VAMC telemedicine site. Consultations were conducted over a live two-way audio and video conferencing system using a high-speed (384 kbps) telecommunication connection. The Milwaukee VAMC used a Tandberg videoconference system with 27-inch Sony monitors; the Iron Mountain VAMC used a VTEL FRED unit with a 27-inch monitor. The established sound and video quality of TM was near perfect, and both parties were able to change the field of view by zooming in or out as needed. The nurse at the Iron Mountain VA assisted the physician during the physical examination. Electronic medical records of each patient were available to the Milwaukee physicians by accessing the VA’s computer network, and an electronic progress note for each consultation was entered into the patient’s medical record.

### In-Person Consultation Process

Patients undergoing IP care at the Milwaukee VAMC are from the Milwaukee metropolitan area. In our study, IP patients were checked in by a clinic nurse and placed in an examination room previously set up with the video recording equipment. Physicians conducted an IP medical interview and a hands-on physical examination with the patients. No nurse was present in the room during these consultations. Physicians had access to electronic medical records of each participating patient and entered an electronic progress note for each consultation.

### Data Collection

For TM examinations, a VCR was used to record the picture and sound as viewed from the TM physician’s TV monitor in Milwaukee. This 27-inch monitor displayed the image of the remote site examination room in Iron Mountain, including a full-body frontal view of the seated patient and an upper body frontal view of the nurse seated behind a desk next to the patient. In addition, a 5-inch diagonal picture-in-picture frontal view image of the physician was projected in the upper-left corner of the monitor. The IP consultations were recorded by a digital camcorder equipped with a wide-angle lens mounted on a tripod placed in the examination room. The camera and microphone were placed in an unobtrusive manner, and both the patient and physician were alerted to the presence of these devices. The camcorder was set up in a standardized fashion, and the resulting image included a full body, oblique view of both the seated patient and the seated physician.

### Measurement of Physician-Patient Communication

We used the Roter Interaction Analysis System (RIAS) to code the medical dialogue. The RIAS treats a complete thought, defined as a simple sentence, a sentence clause, a sentence fragment, or single word, as the unit of analysis. A complete thought may be categorized as one of 38 mutually exclusive and exhaustive codes. Coding is done directly from video recordings without transcription. In the current study, a nurse was always present during the TM consultations, and her communication was coded separately from that of the TM physician. In 8 of the 19 consultations, the patient was accompanied by a companion, and his or her speech was also coded separately. RIAS coding was performed by trained coders under the supervision of Dr. Roter (co-investigator) at Johns Hopkins University. Dr. Roter trained coders in the use of the RIAS System over several weeks, using a coding manual with detailed definitions and annotated examples, and training tapes demonstrating standardized techniques. Inter-rater reliability was calculated based on double coding of 20% sample by 2 independent and blinded coders. In the current study, reliability ranged across categories roughly from 0.7 to 0.9, based on Pearson correlation coefficients.

### Measurement and Analysis of Nonverbal Communication—Global Affect Ratings

In addition to verbal communication, the RIAS is also used for coding nonverbal communication. In this study, we used RIAS for coding of physician and patient global affect ratings. These ratings were based on the emotional tone of each speaker for the following dimensions: (1) angry or irritated, (2) anxious or nervous, (3) depressed or sad (patient only), (4) emotionally distressed (patient only), (5) dominant or assertive, (6) interested or attentive, (7) friendly or warm, (8) responsive or engaged, (9) sympathetic or empathetic, (10) hurried or rushed (physician only), and (11) respectful. The global affect ratings were performed by trained coders under the supervision of co-investigator Dr. Roter at Johns Hopkins University. The coders assigned an overall Likert score (1 = lowest and 6 = highest) for each dimension for the whole interaction. Inter-rater reliability, calculated as percentage agreement by double coding of 20% random sample by 2 independent and blinded coders, ranged from 87% to 100%.

## Results

### Descriptive Analyses of the Visit Dialogue

Visit length is associated with communication quantity (number of utterances) and was measured for each IP and TM consultation. There were no significant differences in the length of consultations (*P* = 0.96), with IP visits averaging 21.9 ± 7.6 minutes compared to 22.2 ± 12 minutes for TM visits. Descriptive analyses of the visit dialogue can be presented in several ways. First, an overall “profile” of the visits in terms of physicians’, nurses’, and patients’ communication is presented in terms of counts of utterances (“utterance” is defined as a statement or complete thought). The total number of utterances (ie, “all talk”) across all participants (physician, patient, nurse, and patient companion) was similar in the two consultation modes (344 ± 170 utterances during IP and 354 ± 233 during TM). During IP visits, there were an equal number of physician utterances (166 ± 80) and patient utterances (166 ± 88). However, during TM visits, physicians accounted for a higher number of utterances (178 ± 118) as compared to patients (142 ± 127). As noted previously, the nurse was present during TM visits only and, on average, contributed 21 utterances (6%) to the dialogue. The largest categories of nurse contribution included orientations (5 utterances), agreements (3 utterances), biomedical information (3 utterances), and closed medical questions (2.5 utterances). A patient companion was equally likely to be present in both IP and TM consultations: 3 of 8 (38%) IP visits and 5 of 11 (45%) TM visits. The verbal contribution of the companion was similar in the two encounter modes, accounting for an average of 7% (31 utterances) of dialogue during IP visits and 9% (28 utterances) during TM visits (*P* = .8). Most of the companion’s contribution to the dialogue was in the provision of information to the physician about the patient’s medical symptoms (12 utterances), therapeutic regimen (4.5 utterances), lifestyle and psychosocial status (3 utterances), and agreements (3 utterances).

### Verbal Dominance Ratio

Descriptive analyses of the visit dialogue can also be presented as various ratios to capture relative amounts of talk. The ratio of total number of provider utterances to total number of patient utterances is called the “verbal dominance ratio” and is a summary measure of “patient-centered” versus “physician-centered” style of communication. A verbal dominance ratio of 1 indicates equal participation by patient and physician and is indicative of a patient-centered interview style, whereas a ratio of greater than 1 indicates a physician-centered interview style. In this study, the physician dominated the interview more during TM as compared to IP visits (TM = 1.45 vs. IP = 1.13; t = -1.25, *P* = .23). When the second provider (ie, the nurse) was included in the provider portion of the ratio calculation, verbal dominance ratio for TM was even higher (1.7). Due to small sample size, the differences (TM vs. IP) in verbal dominance ratios were not statistically significant.

### Analyses of RIAS Content Categories for Physician and Patient Dialogue During TM and IP Visits

For the purposes of this study, each utterance made during the visit by the patient, physician, and nurse was coded into one of 38 mutually exclusive and exhaustive RIAS categories. During analysis, the 38 mutually exclusive content categories are often combined into larger “subsuming categories” that share common meaning [12]. In this study, 38 RIAS content categories were combined into 10 larger subsuming categories. The number of utterances (mean and SD) by the physician and the patient for each subsuming communication category are presented in Table 1.

**Table 1:** Physician-patient verbal communication during TM and IP visits

Speaker	Physician				Patient			
Type of Visit	TM	IP			TM	IP		
RIAS Subsuming Communication Category	N (SD)	N (SD)	t	*P*	N (SD)	N (SD)	t	*P*
Information-gathering, biomedical	29.1 (18.9)	26.5 (23.1)	-0.26	.80	4.9 (4.3)	4.6 (2.4)	-1.84	.86
Information-gathering, psychosocial	4.9 (5.6)	4.8 (4.4)	-0.07	.95	0.2 (0.6)	0.1(0.4)	-0.26	.80
Information-giving, biomedical	71.1 (56.6)	51 (29.8)	-1.00	.33	86.3 (75.8)	97 (58.9)	0.35	.73
Information-giving, psychosocial	1.4 (2.4)	4.8 (9.2)	1.02	.34	11.8 (15.2)	12.8 (17.3)	0.12	.91
Positive talk	31 (23)	27 (23)	-0.36	.73	24 (26)	32 (19)	0.80	.44
Negative talk	0.2 (0.4)	0.4 (0.5)	0.88	.40	1.5 (2)	0.8 (1)	-.98	.34
Social talk	3.7 (3)	2.1 (1.8)	-1.46	.16	2.6 (2.8)	1.3 (1.4)	-1.32	.21
Rapport building/emotional responsiveness	8.1 (9.1)	10.4 (9.8)	0.52	.61	5.4 (5.4)	6.6 (7.2)	0.42	.69
Partnership building	14.2 (11.5)	12.3 (7.5)	-0.44	.66	1.9 (3.1)	2.9 (2.4)	0.76	.46
Orientation statements	9.7 (8.9)	19 (9.4)	2.17	.047	0.9 (1.25)	1.6 (2.7)	0.78	.46

### Information-Gathering, Biomedical and Psychosocial

These RIAS categories include closed- and open-ended questions asked by patient and physician related to (1) medical condition and therapeutic regimen (biomedical), and (2) lifestyle and psychosocial topics. Overall, both patients and physicians engaged in more biomedical than psychosocial information gathering during both TM and IP visits. When comparing the two consultation modes, there was no significant difference in the amount of biomedical information gathered by physicians (TM = 29.1 vs. IP = 26.5; t = -0.26, *P* = .80) or patients (TM =4.9 vs. IP = 4.6; t = -1.84, *P* = .86), or psychosocial information gathered by physicians (TM = 4.9 vs. IP = 4.8; t = -0.07, *P* = .95) or patients (TM = 0.2 vs. IP = 0.1; t = -0.26, *P* = .80).

### Information-Giving, Biomedical and Psychosocial

These RIAS categories include information sharing by patient and information sharing plus counseling by physician related to (1) medical condition and therapeutic regimen (biomedical), and (2) lifestyle and psychosocial topics. It was noted that both physician and patient predominantly exchanged information regarding biomedical versus lifestyle and psychosocial topics during both IP and TM consultations. Physicians provided more patient counseling and information sharing regarding biomedical issues during TM as compared to IP visits (TM = 71.1 vs. IP = 51; t = -1.00, *P* = .33). Conversely, physicians made a greater number of counseling statements and provided more information on psychosocial and lifestyle issues during IP versus TM visits (IP = 4.8 vs. TM = 1.4; t = 1.02, *P* = .34). There was no difference between TM and IP for patient information giving for either biomedical (TM = 86.3 vs. IP = 97; t = 0.35, *P* = .73) or psychosocial (TM = 11.8 vs. IP = 12.8; t = 0.12, *P* = .91) categories. While none of the results in the information-giving category reached statistical significance, the data suggest that physician communication was more patient-centered (more dialogue around psychosocial and lifestyle issues) during IP visits and more physician-centered (more biomedical talk and less psychosocial/lifestyle talk) during TM visits.

### Positive Talk, Negative Talk, and Social Talk

The category “positive talk” includes laughter, agreement, and approval. Positive talk constituted a considerable portion of total talk for both physicians and patients (TM = 17.9%, SD = 5.3; IP = 18%, SD = 4.8), there was no difference in physician talk (TM = 31 vs. IP = 27; t = -0.36, *P* = .73) or patient talk (TM = 24 vs. IP = 32; t = 0.80, *P* = .44) in this category when comparing the two visit types. “Negative talk” includes statements of disagreement and criticism. Such talk was rarely engaged in, and there was no significant difference in this category for physicians (TM = 0.2 vs. IP = 0.4; t = 0.88, *P* = .40) or patients (TM = 1.5 vs. IP = 0.8; t = -0.98, P = .34) when comparing the two visit types. “Social talk” includes all nonmedical dialogue. Physicians and patients use social talk during medical visits to develop rapport and display interest. Social talk was infrequent overall. Although more social talk occurred during TM visits, there was no significant difference in this category for physicians (TM = 3.7 vs. IP = 2.1; t = -1.46, *P* = .16) or patients (TM = 2.6 vs. IP = 1.3; t = -1.32, *P* = .21) when comparing the two visit types.

### Rapport Building/Emotional Responsiveness and Partnership Building

The RIAS category “rapport building/emotional responsiveness” includes instances in which the patient or physician shows concern or asks for or provides an opinion or reassurance. There was no difference between TM and IP visits for physicians (TM = 8.1 vs. IP = 10.4; t = 0.52, *P* = .61) or patients (TM = 5.4 vs. IP = 6.6; t = 0.42, *P* = .69) in this category.  “Partnership building” includes verbal communication that indicates understanding, as well as instances in which the patient or physician paraphrases or interprets the other’s talk. There was no difference in partnership building between TM and IP visits for physicians (TM = 14.2 vs. IP = 12.3; t = -0.44, *P* = .66) or patients (TM = 1.9 vs. IP = 2.9; t = 0.76, *P* = .46).

### Orientation Statements

This category includes physician statements that tell the patient what is expected during the consultation or what is about to happen (eg, “I am going to check your pulse now.”), as well as statements that serve to orient the patient to major topics of discussion or the physical flow of the visit. Physicians used fewer orientation statements during TM (9.7) as compared to IP (19) visits (t = 2.17, *P* = .047). Overall, patients made few orientation statements (eg, requests for instructions related to flow of the visit and physical exam) and there was no difference when comparing the two visit types (TM = 0.9 vs. IP = 1.6; t = 0.78, *P* = .46).

### Requests for Repetition and Unintelligible Utterances

Requests for repetition are statements (eg, “What?” “Come again?” “How is that?” “Would you repeat that?”) in response to instances in which one participant has not clearly heard or understood another’s words or statements. Such requests are indicative of perceptual difficulties. Utterances are coded as “unintelligible” when the coder is unable to understand what a participant (eg, patient or physician) has said. These two categories are not part of the subsuming Roter categories (Table 1) used in this study. However, we coded and analyzed TM vs IP data for these categories to detect any differences in clarity of physician-patient verbal communication, as the effect of TM technology on the quality of communication is an area of concern. Patients made significantly more requests for repetition during TM visits (TM = 1.64 vs. IP = .38; t = -2.33, *P* = .034). Physicians also made more requests for repetition during TM, although the difference was not statistically significant (TM = .45 vs. IP = .00; t = -2.19, *P* = .053). Unintelligible utterances in physician dialogue were more common during IP visits (TM = .27 vs. IP = 3.75; t = 2.66, *P* = .031). There was no significant difference in the number of unintelligible utterances by patients, when comparing the two consultation modes (TM = .91 vs. IP = 2.38; t = 1.16, *P* = .28).

### Nonverbal Communication

The RIAS was used for global ratings of the emotional tone (nonverbal communication) of both the patient and physician. Based on vocal qualities, coders captured the global ratings (ie, a single rating for the entire visit) of emotional affect for both the patient and physician on 11 affective dimensions (anger, anxiety, sadness, distress, dominance, interest, friendliness, responsiveness, sympathy, hurried, and respectful) for each IP and TM visit. There were no significant differences noted in global affect ratings for physicians (TM = 2.4 vs. IP = 2.4; t = -0.15, *P* = .88) or patients (TM = 2.3 vs. IP = 2.2; t = -1.17, *P* = .26) during either type of visit.

## Discussion

A popular conceptual model used to describe physician-patient communication defines communication styles as either “physician-centered” or “patient-centered.” Physician behaviors, such as gathering of information via closed-ended questions, testing hypotheses to make a diagnosis, giving medical directions, and controlling the visit, represent a “physician-centered” style of communication. The patient’s role is to listen, follow the physician’s directions, and play a passive role during the medical encounter. This type of communication is less successful in addressing the needs of the patient [13-15]. Conversely, a “patient-centered” style of communication is characterized by physician behaviors such as asking open-ended questions, partnership building, shared decision making, information sharing, counseling, and using statements of concern, agreement, and approval. The patient’s role is to participate actively in making decisions, to express opinions or concerns about his or her health, and to ask for information. This style is more successful in addressing patient needs and is associated with higher patient satisfaction, better psychosocial adjustment, and improved health outcomes [13-19].

The findings of this study suggest that the use of TM does influence patient-physician communication style. During TM visits, physicians were more likely to dominate the dialogue, as evidenced by a higher verbal dominance ratio (TM = 1.45 vs. IP = 1.13; t = -1.25, *P* = .23). In addition, both physicians and patients were more likely to address biomedical topics during TM visits (topics associated with a physician-centered style of communication), while discussion around psychosocial and lifestyle issues (topics associated with a patient-centered style of communication) was limited. These findings corroborate those reported by Street et al’s [6] study in which content analysis of 26 TM consultations between specialists, primary care physicians, and patients showed that the specialists were the dominant communicators in terms of asking questions and displaying controlling behavior.

Clinical TM consultations fundamentally differ from IP encounters due to the physical separation of physician and patient. Direct physical examination and interview are not possible, and a virtual environment replaces the familiar physician’s office. It is possible that the physical separation and the lack of the hands-on physical examination reinforce a “physician-centered” interview style observed during TM consultations. Whether such differences in communication, particularly in the absence of the hands-on examination, have an effect on quality of care, health outcomes, and future patient utilization of TM is not known.

We observed patients to be less engaged (less talkative) and more likely to take on a passive role during TM as compared to IP visits. It is possible that poor patient participation and communication during TM visits are due to the lack of familiarity with technology and the perception of physician detachment due to the inherent physical separation. Patient concerns about privacy and confidentiality during TM consultations may further inhibit patient participation, especially if the dialogue involves collecting data on sensitive or personal topics (eg, sexual history).

Clinical TM consultations frequently involve a three-party communication exchange between a specialist at a tertiary site, and a patient and second provider at a remote site. In Street’s study, the presence of a referring primary care physician was linked to inhibited patient communication and lack of direct engagement with the consulting physician [6]. While it is possible that the presence of a second provider may result in poor patient participation [6], it is also possible that this presence helps promote confidence during TM, as patients feel they are getting attention from two health care professionals (two physicians or a physician and a nurse) versus only one physician during IP visits [20]. In our study, a trained clinical nurse assisted the patient during each TM consultation. On average, the nurse contributed only 6% of all utterances during the TM visit and was the least verbally active participant. It appears that the presence of a trained nurse provider instead of a referring physician may be less detrimental to communication between the patient and the TM physician.

The present study results suggest that TM visits are less patient centered than IP visits. In contrast, results of studies that use patient self report to measure patient satisfaction often indicate high patient satisfaction with TM [21]. We believe that a number of patient factors may explain these differences between third-party evaluation of communication during TM, as in the present study, and patient self report of satisfaction. Patients, in general, are likely to view their medical care in a favorable light. In addition, it is possible that patient expectations for quality of communication are different when it comes to TM versus IP care (ie, patients may have a lower expectation from TM consultation and therefore be less critical of shortcomings in communication). Convenience of TM may also play a role. A number of studies have reported high patient satisfaction with TM because it is convenient (reduced travel) and improves patient access to specialist physician care [21]. Patients may also have a positive perception of TM due to the use of latest technology, hence promoting confidence that they are receiving highest quality care. Infatuation with the use of technology during medical care has been reported in the TM literature. Baigent et al [22] and Gammon et al [23] found that patients reported enjoying video consultations and were inspired by the use of technology. In addition, the presence of a second provider during TM may be viewed positively by patients, who perceive it as more attention—two health care professionals (two physicians or a physician and a nurse) versus only one physician during IP visits [20].

### Study Limitations

The study is small and exploratory with obvious limitations in terms of experimental design and statistical power. Nevertheless, the study provides a framework for detailed observations and description of patient-physician communication during TM consultations that can be useful in the design of future research in this area. Undoubtedly, the quality of interaction during TM and its potential impact on health care outcomes is an area of growing importance. Further research is needed to help fill the current gaps in the literature and to develop specific interventions that can improve the quality of communication during TM encounters.

## Abbreviations

TM: telemedicine
IP: in-person
VAMC: Veterans Affairs Medical Center
VA: veterans affairs
RIAS: Roter Interaction Analyses System
